# Transcriptomic analysis reveals unique molecular factors for lipid hydrolysis, secondary cell-walls and oxidative protection associated with thermotolerance in perennial grass

**DOI:** 10.1186/s12864-018-4437-z

**Published:** 2018-01-22

**Authors:** Yi Xu, Bingru Huang

**Affiliations:** Department of Plant Biology, Rutgers University, New Brunswick, NJ 08901 USA

**Keywords:** Turfgrass, Heat stress, RNA-seq, Transcriptomic profiling, qRT-PCR

## Abstract

**Background:**

Heat stress is the primary abiotic stress limiting growth of cool-season grass species. The objective of this study was to determine molecular factors and metabolic pathways associated with superior heat tolerance in thermal bentgrass (*Agrostis scabra*) by comparative analysis of transcriptomic profiles with its co-generic heat-sensitive species creeping bentgrass (*A. stolonifera*).

**Results:**

Transcriptomic profiling by RNA-seq in both heat-sensitive *A. stolonifera* (cv. ‘Penncross’) and heat-tolerant *A. scabra* exposed to heat stress found 1393 (675 up- and 718 down-regulated) and 1508 (777 up- and 731 down-regulated) differentially-expressed genes, respectively. The superior heat tolerance in *A. scabra* was associated with more up-regulation of genes in oxidative protection, proline biosynthesis, lipid hydrolysis, hemicellulose and lignin biosynthesis, compared to heat-sensitive *A. stolonifera*. Several transcriptional factors (TFs), such as high mobility group B protein 7 (HMGB7), dehydration-responsive element-binding factor 1a (DREB1a), multiprotein-bridging factor 1c (MBF1c), CCCH-domain containing protein 47 (CCCH47), were also found to be up-regulated in *A. scabra* under heat stress.

**Conclusions:**

The unique TFs and genes identified in thermal *A. scabra* could be potential candidate genes for genetic modification of cultivated grass species for improving heat tolerance, and the associated pathways could contribute to the transcriptional regulation for superior heat tolerance in bentgrass species.

**Electronic supplementary material:**

The online version of this article (10.1186/s12864-018-4437-z) contains supplementary material, which is available to authorized users.

## Background

Heat stress is one of the major environmental stresses limiting plant growth for cool-season plant species. Extensive effort has been taken to investigate physiology and molecular mechanisms of heat tolerance in various plant species (for review, see Wahid et al. [1]). Further studies to determine physiological basis, phenotypic flexibility, and molecular factors modulating plant heat tolerance are essential. Furthermore, it is also imperative to apply genomic, proteomic, and transcriptomic approaches to better understand the molecular basis of plant response to heat stress and heat tolerance.

RNA sequencing has been widely used to investigate plant molecular responses to stress conditions on the scale of the entire transcriptome [2]. The information obtained could further be used to guide plant molecular engineering or marker development. The transcriptomic profiling for heat-responsive genes has been conducted in a large variety of plant species, including model plant species, such as Arabidopsis [3], annual crops, such as rice [4, 5], wheat [6], barley [7], and perennial grass species, such as switchgrass [8] and tall fescue [9]. Previous work on transcriptomic analysis related to heat stress have mainly reported heat-responsive genes involved in various metabolic processes, such as those in respiration (glycolysis and tricarboxylic acid cycle), photosynthesis (light reactions) [4], protein modification [8], antioxidant metabolism [7], and lipid metabolism [10]. In addition, some transcription factor families, such as heat shock factor (HSF), APETALA2/ethylene-responsive element binding factor (AP2/ERF), dehydration-responsive element binding factor (DREB), myeloblastosis factor (MYB), WRKY-domain factor (WRKY), and zinc finger protein, were activated upon heat stress [3, 4, 7, 10, 11]. Although numerous heat-responsive genes have been identified, transcriptional factors and genes uniquely associated with heat tolerance should be further explored for in-depth understanding of molecular mechanisms conferring heat tolerance.

One approach to unraveling mechanisms of plant tolerance to stresses is to examine plants adapted to extremely stressful environments. A temperate (C3) perennial grass species, thermal bentgrass (*A. scabra*) endemic to geothermal areas of Yellowstone National Park, exhibits superior heat tolerance to other C3 grass species, as it is able to survive at soil temperature up to 45 °C [12, 13], while soil temperature over 18 °C or air temperature over 24 °C is detrimental for most C3 grass species [14]. Physiological, proteomic, and metabolic analysis with thermal bentgrass have found that superior heat tolerance of *A. scabra* was associated with the adjustment of various metabolic processes, including lowering respiratory consumption of carbohydrates, increases of alternative respiration and carbon use efficiency [15–18], activation of antioxidant metabolism, induction of stress-protective proteins, such as heat shock proteins [19–21] and the accumulation of osmoprotectants, such as soluble sugars and proline [22]. However, the molecular factors underlying the superior heat tolerance of the thermal grass species are not well documented, but such information is useful for improving heat tolerance in cultivated grass species.

The objective of this study was to identify unique transcriptional factors and genes, as well as the associated metabolic pathways accounting for the superior heat tolerance of the wild grass species, thermal *A. scabra*, by comparative analysis of the transcriptomic changes in response to heat stress between thermal *A. scabra* and its co-generic heat-sensitive species (*A. stolonifera*)*.*

## Methods

### Plant materials and growth conditions

Tillers (30 per individual plant) of *A. stolonifera* (‘Penncross’) or *A. scabra* (‘NTAS’) were collected from stock plants and transferred to plastic containers (57 × 44 × 30 cm, 12 drainage holes) filled with fritted clay medium (Profile Products, Deerfield, IL). Plants were established for 35 d in a greenhouse with average temperature of 23/20 °C (day/night), 60% relative humidity (RH), and 750 μmol m^−2^ s^−1^ photosynthetically active radiation (PAR) from natural sunlight and supplemental lighting. Plants were irrigated daily, fertilized twice per week with half-strength Hoagland’s nutrient solution [23], and trimmed to 2 cm once per week during establishment. Plants were not trimmed during the final week of establishment to allow for sufficient regrowth prior to stress imposition, after which time all plants were transferred to controlled-environment growth chambers (Environmental Growth Chamber, Chagrin Falls, Ohio).

### Heat stress treatments and experimental design

Plants were maintained in controlled-environment growth chambers controlled at 22/18 °C (day/night), 600 μmol m^−2^ s^−1^ PAR, 60% RH, and 14-h photoperiod for one week prior to stress imposition, and then air temperature was raised to 35/30 °C to impose heat stress for 21 d. During stress treatment, plants were irrigated daily, and fertilized twice per week with half-strength Hoagland’s nutrient solution. The experiment was arranged in a split-plot design with temperature treatment (control or heat) as the main plots and grass species (*A. scabra* or *A. stolonifera*) as subplots. Each species was replicated in four containers and each temperature treatment was repeated in four growth chambers. Plants under the same temperature were relocated across growth chambers every 3 d to avoid possible confounding effects of chamber environmental variations.

#### Physiological measurements

Leaf relative water content (RWC), chlorophyll content (Chl) and electrolyte leakage (EL) were measured at 0 and 21 d of heat stress to assess differential physiological responses of the two plant species under both control and heat stress conditions. Approximately 0.8 g fresh leaf tissue was collected from four individual plants per line per container, and then pooled for RWC, EL, and Chl measurements. For RWC, 0.2 g of leaf blades were first weighed for fresh weight (FW), soaked in water for 12 h and again weighed for turgid weight (TW), dried in an oven at 80 °C for 3 d, and finally weighed for dry weight (DW). RWC was calculated using the formula (%) = ([FW - DW] / [TW - DW]) × 100 [24]. For Chl, approximately 0.2 g fresh leaf tissue was submerged in 10 ml dimethyl sulphoxide for 3 d to extract total chlorophyll. The absorbance of the leaf extract was measured at 663 nm and 645 nm with a spectrophotometer (Spectronic Genesys 2; Spectronic Instruments, Rochester, NY) and Chl calculated using the formula described in [25]. For EL, approximately 0.2 g of fresh leaf tissue was rinsed with deionized water, placed in a test tube containing 30 mL deionized water, agitated on a conical shaker for 12 h, and initial conductance (Ci) measured using a conductivity meter (YSI Model 32, Yellow Springs, OH). Tubes containing leaf tissue were then autoclaved at 121 °C for 20 min and again agitated for 12 h. The maximal conductance (C_max_) of incubation solution was then measured and EL (%) was calculated as ((C_i_/C_max_) × 100) [26]. Four biological replicates (*n* = 4) of each species were performed for each parameter under either control or heat stress condition, respectively. Statistical differences between treatment means were separated by Student’s t-test at the P level of 0.05.

### RNA extraction, library preparation, and RNA sequencing

Total RNA was extracted from 200 mg of leaf samples collected at 21 d of heat stress using TRIzol reagent (Life Technologies, Grand Island, NY), then treated with TURBO DNA-free kit (Life Technologies, Grand Island, NY). The quality and quantity of RNA was assessed in a NanoDrop 1000 spectrophotometer (Thermo Fisher Scientific, Waltham, MA). A total of 12 libraries (2 plant species × 2 temperature treatment × 3 biological replicates) were prepared for RNA-seq. Total RNA (2 μg) was used for construction of each library using the Illumina TruSeq RNA Library Prep Kit v2 (Illumina, San Diego, CA) according to the Low Sample (LS) protocol. LS protocol was amended to lower the Elute 2-Fragment-Prime 94 °C incubation time from 8 min to 1 min to generate larger RNA fragments. Indexes were chosen to allow for library multiplexing per run and libraries were pooled in an equimolar fashion. Pooled libraries were prepared for MiSeq run according to Illumina recommendations and loaded into a 600-cycle MiSeq Reagent Kit v3 cartridge (Illumina, San Diego, CA) at a concentration of 20 pM. Each run was set as pair-end (PE) 2 × 300 bp, fastq format only, and no adapter trimming.

### Read alignment, counting, gene expression and functional analysis

Raw reads from MiSeq sequencing were downloaded and analyzed using samtools command flagstat [27]. Reads were then assembled using Trinity [28], with quality trimming using Trimmomatic option. The parameters were set as follows: “Trinity --max_memory 64G, --CPU 8, --bflyCPU 2, --bflyHeapSpaceMax 64G, --trimmomatic ILLUMINACLIP::2:30:15:8:TRUE SLIDINGWINDOW:4:20 LEADING:20 TRAILING:20 MINLEN:60 HEADCROP:6 CROP: 275”. Transcripts obtained were clustered using CDHITEST [29], with the following parameters: “cd-hit-est -c 0.9, −n 8”. The transcripts were then quantified using RSEM [30], which was incorporated as the “align_and_estimate_abundance.pl” script in Trinity program, using default parameters. Differential expression analysis of transcripts were performed using edgeR [31], which was also nested in the “run_DE_analysis.pl” script in Trinity, using default parameters. The ratios of transcript abundances under heat stress to control condition for each species were filtered with threshold of |log2 fold change (log2 FC)| > 1 and false discovery rate (FDR) < 0.01, in order to get differentially expressed genes (DEGs). In addition, the coding regions of transcript assemblies were identified using TransDecoder [28], and then annotated using Trinotate [28], with the options of blastx, blastp, HMMER, signalP, and TMHMM.

Gene ontology (GO) term classification was performed by CateGOrizer [32], using “GO_slim2” method. The GO enrichment analysis for DEGs was performed using GOEAST [33], by first implementing Customized Result Analysis for up- and down-regulated DEGs in each species, respectively, and then comparing between two species in Multi-GOEAST, using default parameters. KEGG pathway enrichment analysis was performed using DAVID v6.8 [34], by using UniProt IDs for the entire transcriptome background and DEGs in both species.

The transcriptome shotgun assembly of both *A. stolonifera* and *A. scabra* were deposited at GenBank Transcriptome Shotgun Assembly (TSA) database, under the accession of GFJH00000000 and GFIW00000000, respectively. The version described in this paper is the first version, GFJH01000000 and GFIW01000000.

### Validation of gene expression levels

Gene expression analysis was performed by quantitative reverse transcriptase polymerase chain reaction (qRT-PCR). Total RNA was isolated from ground leaf powder using TRIzol reagent (Life Technologies, Grand Island, NY) and treated with DNase (TURBO DNA-free kit; Life Technologies, Grand Island, NY) to remove contaminating genomic DNA. Total RNA (2 μg) was reverse-transcribed using a high-capacity cDNA reverse transcription kit (Life Technologies, Grand Island, NY). The synthesized cDNA was amplified in a StepOnePlus Real-Time PCR system (Life Technologies, Grand Island, NY) using the following parameters: pre-heat cycle of 95 °C for 3 min, 40 cycles of 95 °C denaturation for 30 s per cycle, and 60 °C annealing/extension for 30 s per cycle. Power SYBR Green PCR Master Mix (Life Technologies, Grand Island, NY) was the intercalating dye used to detect gene expression level. Gene name, accession number, forward and reverse primer sequences are provided in Table 1. A melting curve analysis was performed for each primer set to confirm its specificity. Actin was used as the reference gene, since its expression was consistent throughout treatments. A ΔΔCt method was used to calculate the relative expression level between genes of interest and reference gene, respectively [35]. Four biological replicates (*n* = 4) from each species were performed for each gene under either control or heat stress condition, respectively. Statistical differences between treatment means were separated by Student’s t-test at the P level of 0.05.

**Table 1:** Primer sequences of genes used in qRT-PCR. Gene names and transcript IDs are also listed

Gene	ID	Primer sequence	
*A. scabra*			
*XET25*	TRINITY_DN127707_c4_g25_i2	Forward	CGACGCTTATCTCCAAACC
		Reverse	GCCATGCCTTGCTCTATC
*GDSL esterase*	TRINITY_DN125263_c6_g4_i1	Forward	CTTCACCAACGGCTACAA
		Reverse	CAGCCCGAGTAGAAGTTTATC
*Dirigent protein 5*	TRINITY_DN89062_c0_g1_i1	Forward	GGACCATCACAGAAGAAAGTAG
		Reverse	CCAGGTTGAAAGAGACATAGTAG
*P5CR*	TRINITY_DN120079_c1_g2_i1	Forward	GGTAAGCGAGACAGGTAAAC
		Reverse	GCGTCCCACGAAATGAA
*Cytochrome P450 77A3*	TRINITY_DN133782_c0_g2_i3	Forward	GATGGATGGACAAGCATCAT
		Reverse	CAGCAGGTTATAGGTACACTTC
*HMGB7*	TRINITY_DN119330_c0_g1_i2	Forward	TGAAGAGGTGGAGGAAGAG
		Reverse	CAGAAACTCTCACACAGAAGAG
*DREB1A*	TRINITY_DN125656_c0_g3_i2	Forward	GCTGTGAGAGTTTCTGGTAAT
		Reverse	AGCTCAGGTCGTTCTACATA
*A. stolonifera*			
*Glycine cleavage system H protein*	TRINITY_DN88310_c1_g1_i3	Forward	ACGGTCGCTGGATAGTATAA
		Reverse	ACGTTCCTGCTCTACTATATCT
*GAPDH A*	TRINITY_DN108728_c4_g45_i1	Forward	CATGGTTCCCTTGACGATT
		Reverse	CCTATGTGATCGGTGTCAAC
*Peroxidase 4*	TRINITY_DN101060_c1_g1_i1	Forward	CGCTTGTCAGACTCTTCTTC
		Reverse	TCCACGGATGGAGCTATT
*Beta-glucosidase 3*	TRINITY_DN113597_c1_g1_i1	Forward	GATGGGCAGCAGAACATAG
		Reverse	GTGCTTGCAGAGAAGGTATAG
*DIVARICATA*	TRINITY_DN89810_c0_g3_i1	Forward	GCCAACCCTCCTCATATAAA
		Reverse	GTCCATAAACTACGGTAGGG
*ACTIN*	Internal reference	Forward	CCTTTTCCAGCCATCTTTCA
		Reverse	GAGGTCCTTCCTGATATCCA

## Results

### Physiological responses to heat stress

**Fig. 1:**
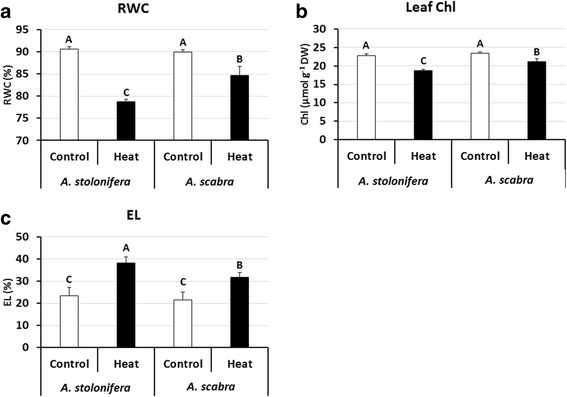
Leaf relative water content (RWC) (**a**), chlorophyll content (Chl) (**b**), and electrolyte leakage (EL) (**c**) of *A. stolonifera* and *A. scabra* under control and heat stress conditions. Data shown are the means of four biological replicates (*n* = 4). Bar represents standard error (SE) for each mean value. Different letters atop bars indicate that significant differences exist at P level < 0.05

Under control conditions, leaf relative water content (RWC) did not differ significantly between *A. stolonifera* and *A. scabra*. Heat treatment caused significantly decline in RWC at 21 d in both *A. stolonifera* and *A. scabra*, by 13.1% and 5.8%, respectively. However, RWC in *A. scabra* was significantly higher than that in *A. stolonifera* (Fig. 1a). No significant differences in leaf chlorophyll content (Chl) were found between *A. stolonifera* and *A. scabra* under control conditions. At 21 d of heat treatment, Chl content decreased significantly in both *A. stolonifera* and *A. scabra*, by 17.8% and 9.6%, respectively; leaf Chl in *A. scabra* was significantly higher than that in *A. stolonifera* (Fig. 1b). For electrolyte leakage (EL), there was no significant difference found between *A. stolonifera* and *A. scabra* under control conditions. Heat stress at 21 d resulted in significantly increases in EL in both *A. stolonifera* and *A. scabra*, by 63.7% and 47.6%, respectively. Leaf EL in *A. scabra* was significantly lower than that in *A. stolonifera* (Fig. 1c).

### Next-generation sequencing of *A. stolonifera* and *A. scabra*

The RNA sequencing yielded more than 19 million reads per library of *A. stolonifera* and *A. scabra* plants exposed to non-stress control and heat stress conditions, providing over 5× coverage of the estimated genome of *A. stolonifera* (Table 2). The de novo transcript assembly by Trinity algorithm had good alignment rate, indicating that the assembled transcripts were largely representing transcriptome in these two species (Table 2). In addition, transcript qualities were also confirmed by long N50 numbers, contig lengths and similar GC contents (Table 3). It is therefore indicated that the Illumina RNA-seq was successfully performed to obtain transcriptional profiles for *A. stolonifera* and *A. scabra* under heat stress.

**Table 2:** RNA-seq overview and read alignment statistics

	*A. stolonifera*	*A. scabra*
Total reads number	19,011,967	19,692,992
Proper pairs	15,699,156 (82.58%)	14,958,404 (75.96%)
Left-only reads	415,178 (2.18%)	451,447 (2.29%)
Right-only reads	841,732 (4.43%)	952,945 (4.84%)
Improper pairs	2,055,901 (10.81%)	3,330,196 (16.91%)

**Table 3:** The de novo transcriptome assembly statistics

	*A. stolonifera*	*A. scabra*
Total assembled bases	417,331,448	450,726,536
Total transcripts	613,045	736,861
N50	996	820
Average contig length	680.75	611.68
GC%	49.66%	49.97%

**Fig. 2:**
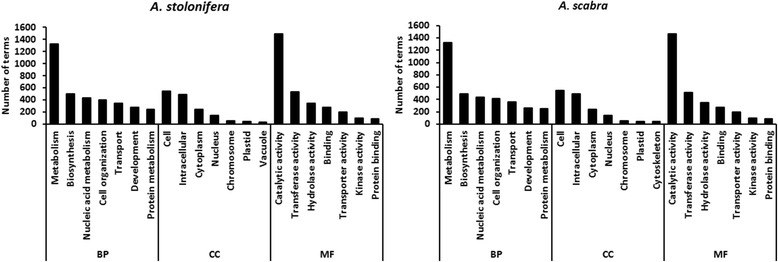
GO term classification of total transcripts in *A. stolonifera* and *A. scabra*. BP: Biological process; MF: Molecular function; CC: Cellular Component

After transcript clustering and annotation, a total of 75,253 and 81,597 UniGenes were obtained by BlastX against NCBI protein NR database (Table 4). Further annotation with GO, KEGG, COG and Pfam also had similar results among them. The components of annotation were mainly from Arabidopsis and rice (Table 5). GO term classification showed that the functional distributions of UniGenes were similar between *A. stolonifera* and *A. scabra* (Fig. 2).

**Table 4:** Number of gene annotations for transcriptome assembly calculated by different databases

	BlastX	GO	KEGG	COG	Pfam
*A. stolonifera*	75,253	62,871	51,968	56,104	34,401
*A. scabra*	81,597	63,816	52,474	56,697	39,856

**Table 5:** Species distribution of gene annotations in transcriptome assembly

	*A. stolonifera*	*A. scabra*
*A. thaliana*	62.93%	62.77%
*O. sativa*	21.47%	21.69%
*Z. mays*	2.35%	2.49%
*H. vulgare*	1.63%	1.55%
*T. aestivum*	1.46%	1.36%
Other	1.30%	1.28%
Unknown	8.86%	8.86%
Total	100%	100%

### GO term enrichment analysis

**Fig. 3:**
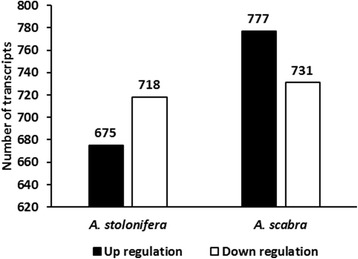
Number of differentially expressed genes (DEGs) under heat stress in A. stolonifera and A. scabra, using the threshold of |log2 fold change (log2 FC)| > 1 and FDR > 0.01

**Fig. 4:**
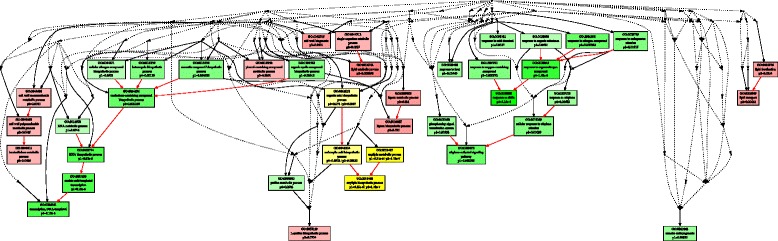
Biological Process (BP) of GO term enrichment for up-regulated DEGs in *A. stolonifera* and *A. scabra*. Green color indicates GO terms that were specifically enriched in *A. stolonifera*. Red color indicates GO terms that were specifically enriched in *A. scabra*. Yellow color indicates GO terms that were commonly enriched in both *A. stolonifera* and *A. scabra*. The density of color was proportional to statistical significance, which was shown as p1 for *P*-value of *A. stolonifera* and p2 for *P*-value of *A. scabra*

**Fig. 5:**
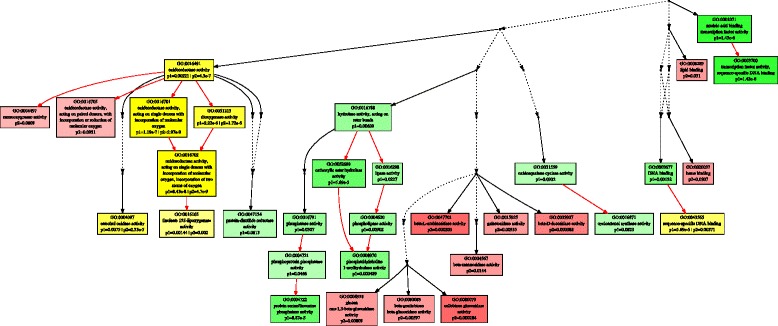
Molecular Function (MF) of GO term enrichment for up-regulated DEGs in *A. stolonifera* and *A. scabra*. Green color indicates GO terms that were specifically enriched in *A. stolonifera*. Red color indicates GO terms that were specifically enriched in *A. scabra*. Yellow color indicates GO terms that were commonly enriched in both *A. stolonifera* and *A. scabra*. The density of color was proportional to statistical significance, which was shown as p1 for *P*-value of *A. stolonifera* and p2 for *P*-value of *A. scabra*

**Fig. 6:**
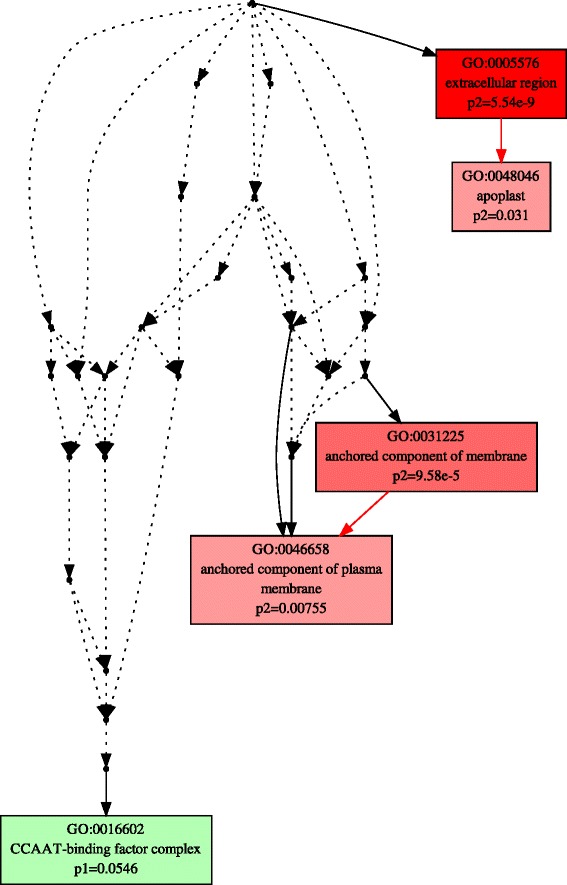
Cellular Component (CC) of GO term enrichment for up-regulated DEGs in *A. stolonifera* and *A. scabra*. Green color indicates GO terms that were specifically enriched in *A. stolonifera*. Red color indicates GO terms that were specifically enriched in *A. scabra*. Yellow color indicates GO terms that were commonly enriched in both *A. stolonifera* and *A. scabra*. The density of color was proportional to statistical significance, which was shown as p1 for *P*-value of *A. stolonifera* and p2 for *P*-value of *A. scabra*

**Fig. 7:**
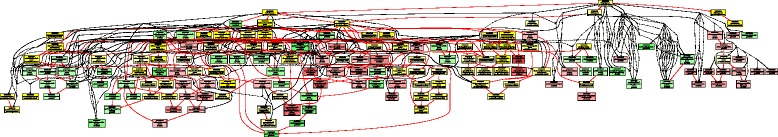
Biological Process (BP) of GO term enrichment for down-regulated DEGs in *A. stolonifera* and *A. scabra*. Green color indicates GO terms that were specifically enriched in *A. stolonifera*. Red color indicates GO terms that were specifically enriched in *A. scabra*. Yellow color indicates GO terms that were commonly enriched in both *A. stolonifera* and *A. scabra*. The density of color was proportional to statistical significance, which was shown as p1 for *P*-value of *A. stolonifera* and p2 for *P*-value of *A. scabra*

**Fig. 8:**
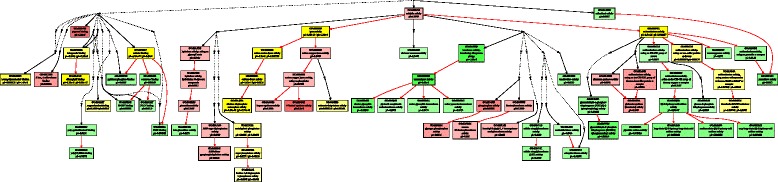
Molecular Function (MF) of GO term enrichment for down-regulated DEGs in *A. stolonifera* and *A. scabra*. Green color indicates GO terms that were specifically enriched in *A. stolonifera*. Red color indicates GO terms that were specifically enriched in *A. scabra*. Yellow color indicates GO terms that were commonly enriched in both *A. stolonifera* and *A. scabra*. The density of color was proportional to statistical significance, which was shown as p1 for *P*-value of *A. stolonifera* and p2 for *P*-value of *A. scabra*

**Fig. 9:**
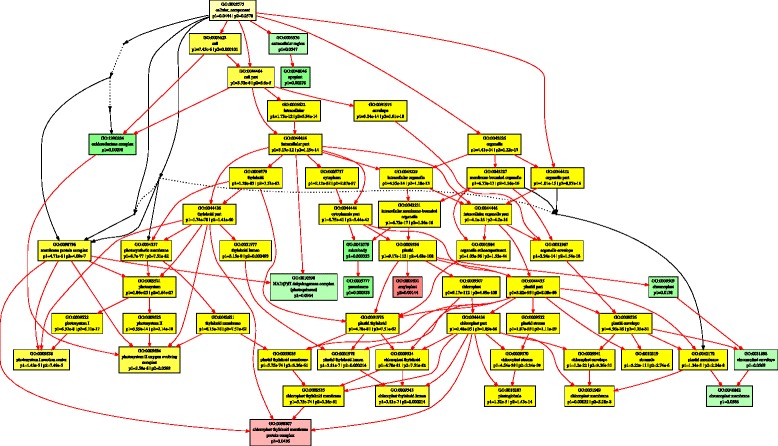
Cellular Component (CC) of GO term enrichment for down-regulated DEGs in *A. stolonifera* and *A. scabra*. Green color indicates GO terms that were specifically enriched in *A. stolonifera*. Red color indicates GO terms that were specifically enriched in *A. scabra*. Yellow color indicates GO terms that were commonly enriched in both *A. stolonifera* and *A. scabra*. The density of color was proportional to statistical significance, which was shown as p1 for *P*-value of *A. stolonifera* and p2 for *P*-value of *A. scabra*

Using the threshold of |log2FC| > 1, and FDR < 0.01, we identified 675 and 777 up-regulated DEGs, and 718 and 731 down-regulated DEGs in *A. stolonifera* and *A. scabra*, respectively, by heat stress (Fig. 3). In order to find out specific molecular factors for the superior heat tolerance in *A. scabra*, up- and down-regulated DEGs due to heat tress were analyzed by GO term enrichment analysis in the two species separately (Figs. 4, 5, 6, 7, 8 and 9; For heat map, see Additional files 1 and 2). In the up-regulated DEGs, several functional categories were enriched only in *A. scabra*, including hemicellulose metabolic process, cell wall biogenesis, L-proline biosynthetic process, lipid catabolic process, lipid transport, lignan biosynthetic process for Biological Process (BP) terms (Fig. 4); In Molecular Function (MF) terms, monooxygenase activity, oxidoreductase activity, several glucosidase activity, and several monosaccharidase activity, such as arabinosidase activity, mannosidase activity, galactosidase activity, fucosidase activity were also uniquely enriched in *A. scabra* (Fig. 5). The uniquely enriched DEGs of *A. scabra* in Cellular Component (CC) terms were mainly at anchored component of membrane and apoplast region (Fig. 6). Some down-regulated DEGs were found to be enriched only in *A. stolonifera*, including DNA-templated transcription, glucose metabolic process, several amino acid metabolic process, such as L-serine, cysteine, and glycine, pentose-phosphate shunt, hydrogen peroxide catabolic process, chloroplast organization, regulation of photosynthesis, positive regulation of translation, and response to oxidative stress in BP terms (Fig. 7). Several cofactor binding functions, such as poly(U) binding, NAD binding, NADP binding, FMN binding, beta-glucosidase activity, cis-trans isomerase activity, several transaminase activity, sulfate adenyltransferase (ATP) activity, adenylate kinase activity, transketolase activity, glyceraldehyde-3-phosphate dehydrogenase (GAPDH) activity, glycolate oxidase activity, glucose-6-phosphate dehydrogenase activity, monooxygenase activity and peroxidase activity were also uniquely enriched in *A. stolonifera* in MF terms (Fig. 8). The CC terms further showed that down-regulated transcripts uniquely enriched in *A. stolonifera* were located in oxidoreductase complex, apoplast, NAD(P)H dehydrogenase complex, peroxisome, and chloroplast membrane (Fig. 9).

The biological process and molecular functions of GO terms in up-regulated DEGs showing specific enrichment to *A. scabra*, and the GO terms in down-regulated DEGs showing specific enrichment to *A. stolonifera* were identified, and the individual transcripts in each category were also analyzed (Tables 6 and 7). In the up-regulated DEGs, those related to cell wall biogenesis, lipid metabolism, proline biosynthesis, lignan biosynthesis, oxidoreductase activity and glucosidase activity, were uniquely enriched in *A. scabra* (Table 6). The down-regulated DEGs found only in *A. stolonifera* included dhurrin biosynthetic process, amino acid metabolism, glucose metabolic process, pentose phosphate shunt, peroxidase activity, beta-glucosidase activity, cis-trans isomerase activity, aminotransferase activity, sulfate adenylyltranferase activity, transketolase activity, Glyceraldehyde 3-phosphate dehydrogenase (GAPDH) activity, glycolate oxidase activity, chloroplast organization, regulation of transcription and translation, energy metabolism and monooxygenase activity (Table 7).

**Table 6:** GO terms in up-regulated DEGs that showed specific enrichment to *A. scabra*

GO ID	Ontology	Term	Level	Transcript ID	Annotation	Log2 FC in *A. scabra*
GO:0042546	biological_process	cell wall biogenesis	2	TRINITY_DN116019_c0_g1	Omega-hydroxypalmitate O-feruloyl transferase	7.48
GO:0010410	biological_process	hemicellulose metabolic process	4	TRINITY_DN127707_c4_g25	XET25	6.60
				TRINITY_DN117099_c7_g1	XET9	6.42
				TRINITY_DN113328_c0_g1	Fasciclin-like arabinogalactan protein 11	4.87
				TRINITY_DN113946_c0_g1	Fasciclin-like arabinogalactan protein 11	4.04
				TRINITY_DN130813_c0_g2	Homeobox protein knotted-1-like 3	3.69
				TRINITY_DN136911_c1_g3	Cellulose synthase A	2.43
				TRINITY_DN132925_c0_g1	COBRA-like protein 7	2.25
				TRINITY_DN122927_c3_g1	COBRA-like protein 5	2.24
				TRINITY_DN121898_c1_g6	Probable glucuronosyltransferase	2.18
				TRINITY_DN121458_c1_g7	XET8	2.00
				TRINITY_DN133427_c0_g2	Microtubule-associated protein 70–4	1.93
				TRINITY_DN134298_c1_g3	Delta(24)-sterol reductase	1.62
				TRINITY_DN117099_c6_g2	XET8	1.33
GO:0016042	biological_process	lipid catabolic process	5	TRINITY_DN125263_c6_g4	GDSL esterase	8.66
				TRINITY_DN118385_c1_g5	Phospholipase A1	7.60
				TRINITY_DN123914_c1_g1	GDSL esterase	6.52
				TRINITY_DN123914_c0_g1	GDSL esterase	5.69
				TRINITY_DN129053_c1_g4	GDSL esterase	5.60
				TRINITY_DN119083_c0_g10	GDSL esterase	5.59
				TRINITY_DN120545_c1_g2	GDSL esterase	5.58
				TRINITY_DN125263_c6_g1	GDSL esterase	5.36
				TRINITY_DN119083_c0_g5	GDSL esterase	5.24
				TRINITY_DN135699_c2_g7	GDSL esterase	5.13
				TRINITY_DN127509_c3_g4	Patatin-like protein 1	4.99
				TRINITY_DN99646_c0_g5	Phospholipase A1	4.29
				TRINITY_DN115628_c0_g1	GDSL esterase	4.18
				TRINITY_DN128761_c1_g2	Patatin-like protein 1	4.06
				TRINITY_DN116954_c1_g1	GDSL esterase	3.62
				TRINITY_DN120720_c3_g15	GDSL esterase	3.43
				TRINITY_DN133700_c2_g80	Phospholipase A1	3.32
				TRINITY_DN120720_c3_g13	GDSL esterase	3.17
				TRINITY_DN119289_c2_g1	GDSL esterase	2.65
				TRINITY_DN121510_c0_g2	GDSL esterase	1.67
				TRINITY_DN128701_c2_g1	GDSL esterase	1.64
				TRINITY_DN132161_c0_g2	Phospholipase A1	1.57
				TRINITY_DN123294_c0_g3	GDSL esterase	1.48
GO:0018958	biological_process	phenol-containing compound metabolic process	4	TRINITY_DN89062_c0_g1	Dirigent protein 5	4.30
GO:0009807	biological_process	lignan biosynthetic process	8	TRINITY_DN104484_c1_g1	Aureusidin synthase 1	4.04
				TRINITY_DN120244_c9_g1	(+)-larreatricin hydroxylase 1	1.79
GO:0055129	biological_process	L-proline biosynthetic process	10	TRINITY_DN120079_c1_g2	Pyrroline-5-carboxylate reductase	1.82
				TRINITY_DN130046_c0_g1	Pyrroline-5-carboxylate reductase	1.55
				TRINITY_DN122160_c0_g6	Pyrroline-5-carboxylate reductase	1.52
				TRINITY_DN131421_c3_g6	Pyrroline-5-carboxylate reductase	1.28
GO:0006869	biological_process	lipid transport	5	TRINITY_DN125947_c0_g1	Non-specific lipid-transfer protein 4	6.57
GO:0008289	molecular_function	lipid binding	1	TRINITY_DN135528_c2_g5	Non-specific lipid-transfer protein 2B	5.14
				TRINITY_DN87604_c1_g1	Non-specific lipid-transfer protein 41	3.83
				TRINITY_DN102031_c0_g5	Non-specific lipid-transfer protein	3.03
				TRINITY_DN127764_c3_g2	Non-specific lipid-transfer protein 2B	2.78
				TRINITY_DN95559_c0_g1	Non-specific lipid-transfer protein 2G	2.75
				TRINITY_DN127764_c3_g1	Non-specific lipid-transfer protein 2B	1.57
				TRINITY_DN130844_c0_g2	Acyl-CoA-binding domain-containing protein 4	1.43
				TRINITY_DN112894_c3_g4	Non-specific lipid-transfer protein 41	1.41
				TRINITY_DN112894_c3_g6	Non-specific lipid-transfer protein 41	1.07
GO:0004497	molecular_function	monooxygenase activity	1	TRINITY_DN117000_c0_g3	Protochlorophyllide-dependent translocon component 52	7.12
GO:0016705	molecular_function	oxidoreductase activity	1	TRINITY_DN134046_c0_g6	Cytochrome P450 89A2	6.98
				TRINITY_DN133782_c0_g2	Cytochrome P450 77A3	6.45
GO:0020037	molecular_function	heme binding	2	TRINITY_DN134164_c0_g6	Cytochrome P450 89A2	6.36
				TRINITY_DN99451_c0_g1	Indole-3-pyruvate monooxygenase YUCCA11	6.21
				TRINITY_DN127254_c0_g1	Cytochrome P450 86A4	6.05
				TRINITY_DN134164_c0_g7	Cytochrome P450 89A2	5.89
				TRINITY_DN129074_c0_g1	Cytochrome P450 94C1	5.78
				TRINITY_DN120580_c1_g2	Cytochrome P450 86A22	5.69
				TRINITY_DN103063_c0_g1	Cytochrome P450 75A3	5.67
				TRINITY_DN124228_c3_g1	Cytochrome P450 94C1	5.39
				TRINITY_DN116183_c0_g6	Protochlorophyllide-dependent translocon component 52	5.26
				TRINITY_DN125214_c0_g1	Cytochrome P450 71D312	4.71
				TRINITY_DN124182_c0_g3	Alkane hydroxylase MAH1	4.22
				TRINITY_DN126873_c0_g2	Cytochrome P450 78A6	3.92
				TRINITY_DN123592_c1_g1	Cytochrome P450 70B3	3.62
				TRINITY_DN136599_c1_g5	3,9-dihydroxypterocarpan 6A–monooxygenase	2.59
				TRINITY_DN135842_c0_g6	Flavonoid 3′-monooxygenase	2.48
				TRINITY_DN121742_c2_g6	Isoflavone 2′-hydroxylase	2.05
				TRINITY_DN116991_c0_g2	Trans-cinnamate 4-monooxygenase	1.95
				TRINITY_DN129987_c5_g80	Methylsterol monooxygenase 1–2	1.91
				TRINITY_DN132664_c1_g1	Cytochrome P450 90A1	1.86
				TRINITY_DN114050_c0_g2	Methylsterol monooxygenase 1–2	1.36
GO:0004338	molecular_function	glucan exo-1,3-beta-glucosidase activity	1	TRINITY_DN129035_c0_g2	Alpha-galactosidase	7.29
GO:0004567	molecular_function	beta-mannosidase activity	1	TRINITY_DN133682_c0_g9	Beta-glucosidase 7	5.58
GO:0015925	molecular_function	galactosidase activity	1	TRINITY_DN132224_c1_g7	Beta-glucosidase 7	5.07
GO:0033907	molecular_function	beta-D-fucosidase activity	1	TRINITY_DN128665_c0_g1	Beta-glucosidase 8	3.16
GO:0047701	molecular_function	beta-L-arabinosidase activity	1	TRINITY_DN131596_c0_g2	Beta-glucosidase 26	3.06
GO:0080079	molecular_function	cellobiose glucosidase activity	1	TRINITY_DN137358_c2_g12	Galactinol-sucrose galactosyltransferase 2	2.91
GO:0080083	molecular_function	beta-gentiobiose beta-glucosidase activity	1	TRINITY_DN130314_c0_g7	Beta-glucosidase 8	2.84
				TRINITY_DN129035_c0_g1	Alpha-galactosidase	2.61
				TRINITY_DN131467_c0_g5	Beta-glucosidase 9	2.19

The transcriptional regulations under heat stress, log2 fold change (log2 FC), in these GO terms are also listed

**Table 7:** GO terms in down-regulated DEGs that showed specific enrichment to *A. stolonifera*

GO ID	Ontology	Term	Level	ID	Annotation	Log2 FC in *A. stolonifera*
GO:0010132	biological_process	dhurrin biosynthetic process	10	TRINITY_DN102712_c0_g3	4-hydroxyphenylacetaldehyde oxime monooxygenase	−2.23
				TRINITY_DN101496_c0_g2	4-hydroxyphenylacetaldehyde oxime monooxygenase	−2.35
				TRINITY_DN107273_c0_g1	Cyanohydrin beta-glucosyltransferase	−2.52
GO:0006535	biological_process	cysteine biosynthetic process from serine	10	TRINITY_DN111995_c9_g3	Serine hydroxymethyltransferase 1	−1.36
				TRINITY_DN108518_c2_g18	Cysteine synthase	−1.45
GO:0006563	biological_process	L-serine metabolic process	6	TRINITY_DN112384_c6_g19	Serine hydroxymethyltransferase 1	−1.53
				TRINITY_DN111995_c9_g15	Serine hydroxymethyltransferase 1	−1.55
GO:0006544	biological_process	glycine metabolic process	6	TRINITY_DN114224_c3_g11	Cysteine synthase	−1.76
				TRINITY_DN111631_c4_g1	Serine acetyltransferase 2	−1.79
GO:0030170	molecular_function	pyridoxal phosphate binding	2	TRINITY_DN111901_c2_g2	Serine acetyltransferase 2	−1.83
				TRINITY_DN108545_c1_g2	Cysteine synthase	−2.00
				TRINITY_DN111995_c9_g3	Serine hydroxymethyltransferase 1	−1.36
				TRINITY_DN112384_c6_g19	Serine hydroxymethyltransferase 1	−1.53
				TRINITY_DN111995_c9_g15	Serine hydroxymethyltransferase 1	−1.55
				TRINITY_DN104761_c8_g8	Glutamate--glyoxylate aminotransferase 1	−1.86
				TRINITY_DN105135_c11_g1	Glutamate--glyoxylate aminotransferase 1	−1.91
				TRINITY_DN108881_c1_g1	Aminomethyltransferase	−2.03
				TRINITY_DN89720_c3_g1	Glycine cleavage system H protein	−2.30
				TRINITY_DN88310_c1_g1	Glycine cleavage system H protein	−2.49
GO:0004345	molecular_function	glucose-6-phosphate dehydrogenase activity	1	TRINITY_DN106237_c1_g1	Glucose-6-phosphate 1-dehydrogenase 1	−1.71
GO:0006006	biological_process	glucose metabolic process	3	TRINITY_DN110605_c3_g2	Glucose-6-phosphate 1-dehydrogenase 1	−1.73
GO:0050661	molecular_function	NADP binding	2	TRINITY_DN104972_c0_g1	Phosphoglucomutase	−1.73
				TRINITY_DN117464_c1_g8	Glucose-6-phosphate 1-dehydrogenase 1	−1.74
				TRINITY_DN107111_c2_g2	Glucose-6-phosphate 1-dehydrogenase 1	−1.94
				TRINITY_DN92321_c0_g3	Glucose-6-phosphate 1-dehydrogenase 1	−1.97
				TRINITY_DN110467_c12_g1	Glyceraldehyde-3-phosphate dehydrogenase A	−2.59
				TRINITY_DN108728_c4_g8	Glyceraldehyde-3-phosphate dehydrogenase B	−2.78
				TRINITY_DN110467_c13_g13	Glyceraldehyde-3-phosphate dehydrogenase A	−2.90
				TRINITY_DN108728_c4_g45	Glyceraldehyde-3-phosphate dehydrogenase A	−3.09
GO:0006098	biological_process	pentose-phosphate shunt	11	TRINITY_DN106239_c5_g3	Photosystem II stability/assembly factor HCF136	−1.34
				TRINITY_DN104836_c5_g8	Glutamine synthetase	−1.37
				TRINITY_DN115697_c0_g2	Acetyltransferase NSI	−1.63
				TRINITY_DN103999_c8_g1	Glutamine synthetase	−1.64
				TRINITY_DN106237_c1_g1	Glucose-6-phosphate 1-dehydrogenase 1	−1.71
				TRINITY_DN110605_c3_g2	Glucose-6-phosphate 1-dehydrogenase 1	−1.73
				TRINITY_DN111792_c1_g5	Ribulose-phosphate 3-epimerase	−1.86
				TRINITY_DN107111_c2_g2	Glucose-6-phosphate 1-dehydrogenase 1	−1.94
				TRINITY_DN92321_c0_g3	Glucose-6-phosphate 1-dehydrogenase 1	−1.97
				TRINITY_DN111792_c1_g12	Ribulose-phosphate 3-epimerase	−2.01
				TRINITY_DN115720_c2_g1	Ribose-5-phosphate isomerase 3	−2.20
GO:0004601	molecular_function	peroxidase activity	2	TRINITY_DN110325_c0_g1	Uncharacterized protein At1g32220, chloroplastic	−1.11
GO:0006979	biological_process	response to oxidative stress	1	TRINITY_DN114953_c0_g1	UV-B-induced protein At3g17800, chloroplastic	−1.22
GO:0042744	biological_process	hydrogen peroxide catabolic process	3	TRINITY_DN98249_c0_g3	Thioredoxin F	−1.22
				TRINITY_DN100788_c0_g1	Glyoxylate/succinic semialdehyde reductase 1	−1.34
				TRINITY_DN115073_c1_g1	Cryptochrome-1	−1.36
				TRINITY_DN97981_c0_g1	Phospholipid hydroperoxide glutathione peroxidase 1	−1.40
				TRINITY_DN94618_c1_g1	Protein CHLOROPLAST ENHANCING STRESS TOLERANCE, chloroplastic	−1.50
				TRINITY_DN98459_c0_g1	Chromophore lyase CRL	−1.54
				TRINITY_DN95499_c1_g3	Phospholipid hydroperoxide glutathione peroxidase 1	−1.55
				TRINITY_DN101976_c5_g1	Photosynthetic NDH subunit of subcomplex B 5	−1.58
				TRINITY_DN106585_c0_g6	Thioredoxin reductase NTRC	−1.59
				TRINITY_DN102867_c2_g1	Peroxidase 50	−1.62
				TRINITY_DN114075_c0_g1	BTB/POZ and TAZ domain-containing protein 3	−1.81
				TRINITY_DN106929_c2_g1	BTB/POZ and TAZ domain-containing protein 2	−1.91
				TRINITY_DN100903_c0_g1	Thylakoid lumenal 29 kDa protein	−1.96
				TRINITY_DN100475_c0_g1	Thylakoid lumenal 29 kDa protein	−2.10
				TRINITY_DN115590_c0_g1	BTB/POZ and TAZ domain-containing protein 4	−2.16
				TRINITY_DN106093_c0_g1	Peroxidase	−2.58
				TRINITY_DN113217_c1_g1	BTB/POZ and TAZ domain-containing protein 2	−2.65
				TRINITY_DN107140_c0_g4	Thioredoxin-like 3–1	−2.84
				TRINITY_DN113511_c1_g1	Peroxidase 2	−3.76
				TRINITY_DN98542_c3_g1	Peroxidase 54	−4.02
				TRINITY_DN96923_c1_g3	Cationic peroxidase SPC4	−4.91
				TRINITY_DN95139_c0_g1	Cationic peroxidase SPC4	−6.29
				TRINITY_DN100813_c0_g3	Peroxidase 4	−6.93
				TRINITY_DN101060_c1_g1	Peroxidase 4	−9.13
GO:0008422	molecular_function	beta-glucosidase activity	1	TRINITY_DN110778_c2_g6	Glucan endo-1,3-beta-glucosidase 8	−1.64
				TRINITY_DN109548_c0_g2	Beta-glucosidase 10	−1.98
				TRINITY_DN112637_c0_g6	Glucan endo-1,3-beta-glucosidase 11	−2.79
				TRINITY_DN109199_c4_g1	Beta-glucosidase 33	−2.87
				TRINITY_DN115749_c2_g4	Beta-glucosidase 3	−3.89
				TRINITY_DN106744_c2_g2	Beta-glucosidase 10	−4.30
				TRINITY_DN106744_c2_g5	Beta-glucosidase 12	−4.45
				TRINITY_DN98601_c0_g1	Glucan endo-1,3-beta-glucosidase 13	−4.75
				TRINITY_DN107872_c3_g1	Glucan endo-1,3-beta-glucosidase 13	−6.01
				TRINITY_DN108629_c1_g9	Beta-glucosidase 3	−7.87
				TRINITY_DN113597_c1_g1	Beta-glucosidase 3	−8.22
GO:0016859	molecular_function	cis-trans isomerase activity	1	TRINITY_DN102435_c1_g1	Peptidyl-prolyl cis-trans isomerase FKBP16–3	−1.19
				TRINITY_DN96729_c0_g2	Peptidyl-prolyl cis-trans isomerase FKBP17–2	−1.65
				TRINITY_DN94270_c0_g2	Peptidyl-prolyl cis-trans isomerase FKBP18	−2.02
				TRINITY_DN97784_c1_g1	Peptidyl-prolyl cis-trans isomerase FKBP16–4	−2.02
				TRINITY_DN107032_c0_g1	Peptidyl-prolyl cis-trans isomerase CYP38	−2.13
				TRINITY_DN98688_c1_g2	Beta-carotene isomerase D27	−2.42
				TRINITY_DN105648_c0_g1	Peptidyl-prolyl cis-trans isomerase CYP37	−2.51
				TRINITY_DN100063_c0_g1	Peptidyl-prolyl cis-trans isomerase CYP26–2	−2.88
				TRINITY_DN103314_c0_g1	Peptidyl-prolyl cis-trans isomerase CYP37	−3.02
GO:0004760	molecular_function	serine-pyruvate transaminase activity	1	TRINITY_DN104761_c8_g8	Glutamate--glyoxylate aminotransferase 1	−1.86
GO:0008453	molecular_function	alanine-glyoxylate transaminase activity	1	TRINITY_DN105135_c11_g1	Glutamate--glyoxylate aminotransferase 1	−1.91
GO:0047958	molecular_function	glycine:2-oxoglutarate aminotransferase activity	1	TRINITY_DN108610_c12_g11	Serine--glyoxylate aminotransferase	−1.95
				TRINITY_DN108610_c12_g18	Serine--glyoxylate aminotransferase	−2.25
GO:0050281	molecular_function	serine-glyoxylate transaminase activity	1			
GO:0004781	molecular_function	sulfate adenylyltransfer-ase (ATP) activity	1	TRINITY_DN99919_c0_g1	ATP sulfurylase 4	−1.47
				TRINITY_DN111733_c0_g1	ATP sulfurylase 2	−2.58
				TRINITY_DN111633_c0_g6	ATP sulfurylase 2	−2.64
GO:0004017	molecular_function	adenylate kinase activity	2	TRINITY_DN100455_c0_g1	Adenylate kinase 2	−1.35
				TRINITY_DN103594_c0_g8	Adenylate kinase 5	−1.57
GO:0006354	biological_process	DNA-templated transcription, elongation	12	TRINITY_DN106267_c0_g4	Adenylate kinase 5	−1.68
				TRINITY_DN106135_c0_g4	Adenylate kinase 5	−1.72
				TRINITY_DN106106_c0_g1	Adenylate kinase 5	−1.87
GO:0004802	molecular_function	transketolase activity	1	TRINITY_DN117305_c5_g24	Transketolase	−1.39
				TRINITY_DN117516_c6_g24	Transketolase	−1.46
				TRINITY_DN117305_c5_g8	Transketolase	−1.81
GO:0047100	molecular_function	glyceraldehyde-3-phosphate dehydrogenase (NADP+) (phosphorylating) activity	1	TRINITY_DN110467_c12_g1	Glyceraldehyde-3-phosphate dehydrogenase A, chloroplastic	−2.59
				TRINITY_DN108728_c4_g8	Glyceraldehyde-3-phosphate dehydrogenase B, chloroplastic	−2.78
				TRINITY_DN110467_c13_g13	Glyceraldehyde-3-phosphate dehydrogenase A, chloroplastic	−2.90
				TRINITY_DN108728_c4_g45	Glyceraldehyde-3-phosphate dehydrogenase B, chloroplastic	−3.09
GO:0008891	molecular_function	glycolate oxidase activity	1	TRINITY_DN101527_c7_g10	Peroxisomal (S)-2-hydroxy-acid oxidase GLO5	−1.21
GO:0009854	biological_process	oxidative photosynthetic carbon pathway	4	TRINITY_DN101527_c7_g6	Peroxisomal (S)-2-hydroxy-acid oxidase GLO5	−1.29
GO:0010109	biological_process	regulation of photosynthesis	4	TRINITY_DN110877_c7_g22	Glycerate dehydrogenase HPR, peroxisomal	−1.63
GO:0019048	biological_process	modulation by virus of host morphology or physiology	3	TRINITY_DN103470_c9_g13	Peroxisomal (S)-2-hydroxy-acid oxidase GLO1	−1.64
GO:0052852	molecular_function	very-long-chain-(S)-2-hydroxy-acid oxidase activity	1	TRINITY_DN108661_c4_g28	Glycerate dehydrogenase HPR, peroxisomal	−1.75
GO:0052853	molecular_function	long-chain-(S)-2-hydroxy-long-chain-acid oxidase activity	1	TRINITY_DN104606_c11_g2	Peroxisomal (S)-2-hydroxy-acid oxidase GLO1	−1.79
GO:0052854	molecular_function	medium-chain-(S)-2-hydroxy-acid oxidase activity	1	TRINITY_DN101527_c7_g4	Peroxisomal (S)-2-hydroxy-acid oxidase GLO5	−2.94
GO:0009658	biological_process	chloroplast organization	2	TRINITY_DN112733_c1_g4	Inner membrane protein PPF-1, chloroplastic	−1.08
GO:0010027	biological_process	thylakoid membrane organization	4	TRINITY_DN120021_c4_g4	Cytochrome c biogenesis protein CCS1, chloroplastic	−1.24
GO:0043623	biological_process	cellular protein complex assembly	6	TRINITY_DN103959_c1_g2	Zinc finger protein CONSTANS-LIKE 5	−1.27
				TRINITY_DN113749_c1_g2	Plastidal glycolate/glycerate translocator 1, chloroplastic	−1.33
				TRINITY_DN106239_c5_g3	Photosystem II stability/assembly factor HCF136	−1.34
				TRINITY_DN94274_c0_g1	Sec-independent protein translocase protein TATC, chloroplastic	−1.44
				TRINITY_DN94618_c1_g1	Protein CHLOROPLAST ENHANCING STRESS TOLERANCE, chloroplastic	−1.50
				TRINITY_DN100252_c1_g2	Protein THYLAKOID FORMATION1, chloroplastic	−1.53
				TRINITY_DN98459_c0_g1	Chromophore lyase CRL	−1.54
				TRINITY_DN106585_c0_g6	Thioredoxin reductase NTRC	−1.59
				TRINITY_DN106529_c0_g1	Preprotein translocase subunit SECY, chloroplastic	−1.76
GO:0034051	biological_process	negative regulation of plant-type hypersensitive response	8	TRINITY_DN82813_c0_g2	RPM1-interacting protein 4	−1.54
				TRINITY_DN104137_c0_g2	Protein LSD1	−2.48
				TRINITY_DN105439_c0_g2	Protein LSD1	−3.18
				TRINITY_DN105439_c0_g1	Protein LSD1	−3.93
GO:0034250	biological_process	positive regulation of cellular amide metabolic process	7	TRINITY_DN107506_c3_g2	Chloroplast stem-loop binding protein of 41 kDa a, chloroplastic	−1.67
GO:0045727	biological_process	positive regulation of translation	12	TRINITY_DN109357_c3_g14	Chloroplast stem-loop binding protein of 41 kDa b, chloroplastic	−2.81
				TRINITY_DN109357_c3_g9	Chloroplast stem-loop binding protein of 41 kDa b, chloroplastic	−2.87
				TRINITY_DN109357_c3_g19	Chloroplast stem-loop binding protein of 41 kDa b, chloroplastic	−4.39
GO:0008266	molecular_function	poly(U) RNA binding	2	TRINITY_DN118068_c3_g9	31 kDa ribonucleoprotein, chloroplastic	−1.01
				TRINITY_DN111995_c9_g3	Serine hydroxymethyltransferase 1	−1.36
				TRINITY_DN112384_c6_g19	Serine hydroxymethyltransferase 1	−1.53
				TRINITY_DN107506_c3_g2	Chloroplast stem-loop binding protein of 41 kDa a, chloroplastic	−1.67
GO:0051287	molecular_function	NAD binding	2	TRINITY_DN104957_c4_g1	Cytosolic isocitrate dehydrogenase [NADP]	−1.02
				TRINITY_DN100788_c0_g1	Glyoxylate/succinic semialdehyde reductase 1	−1.34
				TRINITY_DN111312_c1_g3	NAD-dependent malic enzyme 1, mitochondrial	−1.42
				TRINITY_DN111312_c0_g2	NAD-dependent malic enzyme 59 kDa isoform, mitochondrial	−1.51
				TRINITY_DN110877_c7_g22	Glycerate dehydrogenase HPR, peroxisomal	−1.63
				TRINITY_DN108661_c4_g28	Glycerate dehydrogenase HPR, peroxisomal	−1.75
				TRINITY_DN110977_c0_g2	Isocitrate dehydrogenase [NADP]	−1.78
GO:0010181	molecular_function	FMN binding	4	TRINITY_DN101527_c7_g10	Peroxisomal (S)-2-hydroxy-acid oxidase GLO5	−1.21
				TRINITY_DN86676_c0_g1	NAD(P)H dehydrogenase (quinone) FQR1	−1.27
				TRINITY_DN101527_c7_g6	Peroxisomal (S)-2-hydroxy-acid oxidase GLO5	−1.29
				TRINITY_DN106478_c0_g2	Putative 12-oxophytodienoate reductase 11	−1.35
GO:0004497	molecular_function	monooxygenase activity	1	TRINITY_DN95871_c1_g1	Zeaxanthin epoxidase, chloroplastic	−1.26
				TRINITY_DN112129_c0_g3	Flavonoid 3′-monooxygenase	−1.54
				TRINITY_DN109168_c1_g3	3,9-dihydroxypterocarpan 6A–monooxygenase	−1.80
				TRINITY_DN95695_c0_g5	Premnaspirodiene oxygenase	−1.96
				TRINITY_DN102712_c0_g3	4-hydroxyphenylacetaldehyde oxime monooxygenase	−2.23
				TRINITY_DN117628_c13_g163	Ribulose bisphosphate carboxylase small chain PW9, chloroplastic	−2.31
				TRINITY_DN108893_c0_g1	Cytochrome P450 711A1	−2.34
				TRINITY_DN101496_c0_g2	4-hydroxyphenylacetaldehyde oxime monooxygenase	−2.35
				TRINITY_DN117628_c13_g182	Ribulose bisphosphate carboxylase small chain PWS4.3, chloroplastic	−2.77
				TRINITY_DN111951_c3_g11	Ribulose bisphosphate carboxylase small chain PWS4.3, chloroplastic	−2.94
				TRINITY_DN117628_c13_g29	Ribulose bisphosphate carboxylase small chain PW9, chloroplastic	−3.12
				TRINITY_DN117628_c13_g288	Ribulose bisphosphate carboxylase small chain PWS4.3, chloroplastic	−3.32
				TRINITY_DN112168_c0_g2	Cytochrome P450 709B2	−3.35
				TRINITY_DN102506_c1_g1	Flavin-containing monooxygenase FMO GS-OX-like 5	−3.45
				TRINITY_DN117628_c13_g145	Ribulose bisphosphate carboxylase small chain PWS4.3, chloroplastic	−3.65
				TRINITY_DN117628_c13_g315	Ribulose bisphosphate carboxylase small chain, chloroplastic	−3.96

The transcriptional regulations under heat stress, log2 fold change (log2 FC), in these GO terms are also listed

### KEGG pathway enrichment analysis

KEGG pathway enrichment analysis compared DEGs between *A. stolonifera* and *A. scabra*, and identified pathways in the degree of enrichment upon heat stress (Tables 8 and 9). In the up-regulated DEGs by heat stress, the top six enriched pathways in *A. scabra* were cutin, suberine and wax biosynthesis, biosynthesis of secondary metabolites, metabolic pathways, fatty acid elongation, phenylpropanoid biosynthesis, ABC transporters, and those in *A. stolonifera* were biosynthesis of secondary metabolites, arginine and proline metabolism, alpha-linolenic acid metabolism, galactose metabolism, beta-alanine metabolism and plant-pathogen interaction (Table 8). In the down-regulated DEGs, the top six enriched pathways were the same in both *A. stolonifera* and *A. scabra*, including metabolic pathways, biosynthesis of secondary metabolites, glyoxylate and dicarboxylate metabolism, carbon metabolism, glycine, serine and threonine metabolism and biosynthesis of antibiotics (Table 9).

**Table 8:** Ranking of KEGG pathway enrichment in up-regulated DEGs between *A. stolonifera* and *A. scabra* under heat stress

Rank	*A. stolonifera*	*A. scabra*
1	Biosynthesis of secondary metabolites	Cutin, suberine and wax biosynthesis
2	Arginine and proline metabolism	Biosynthesis of secondary metabolites
3	alpha-Linolenic acid metabolism	Metabolic pathways
4	Galactose metabolism	Fatty acid elongation
5	beta-Alanine metabolism	Phenylpropanoid biosynthesis
6	Plant-pathogen interaction	ABC transporters

**Table 9:** Ranking of KEGG pathway enrichment in down-regulated DEGs between *A. stolonifera* and *A. scabra* under heat stress

Rank	*A. stolonifera*	*A. scabra*
1	Metabolic pathways	Metabolic pathways
2	Biosynthesis of secondary metabolites	Carbon metabolism
3	Carbon metabolism	Glyoxylate and dicarboxylate metabolism
4	Glyoxylate and dicarboxylate metabolism	Biosynthesis of antibiotics
5	Biosynthesis of antibiotics	Biosynthesis of secondary metabolites
6	Glycine, serine and threonine metabolism	Glycine, serine and threonine metabolism

### Transcription factors related to heat tolerance

Transcription factors (TFs) responsive to heat stress showed high similarity between *A. stolonifera* and *A. scabra*, including up-regulation of ABA-inducible, basic Helix-Loop-Helix (bHLH), ethylene-responsive factor (ERF), protein FD, G-box-binding factor, heat stress factor (HSF), homeobox-leucine zipper, MYB, NAC, nuclear transcription factor Y, WRKY, and down-regulation of APG, PHL1-like, RNA-polymerase sigma factor, zinc-finger protein (Table 10). However, some TFs were uniquely regulated by heat stress in *A. scabra*, such as the up-regulation of high mobility group B protein 7 (HMGB7), dehydration-responsive element-binding factor 1A (DREB1A), multiprotein-bridging factor 1c, CCCH-domain containing protein 47, and down-regulation of GLK1, GATA transcription factor 21 and 26, protein REVEILLE, ASR3, HY5 (Table 11).

**Table 10:** Number of transcription factors differentially expressed in *A. stolonifera* and *A. scabra* under heat stress

Name	*A. stolonifera*		*A. scabra*	
	Up	Down	Up	Down
ABA-inducible	1	0	1	0
APG	0	1	0	1
bHLH	4	1	12	0
Ethylene-responsive	13	1	7	0
Protein FD	1	0	1	0
G-box binding	1	0	2	0
Heat stress factor	1	0	3	0
Homeobox-leucine zipper	4	0	5	0
MYB/MYC	1	1	0	1
NAC	5	1	2	1
Nuclear transcription factor Y	4	1	2	0
PHL1-like	0	1	0	1
Scarecrow-like	1	1	1	0
RNA polymerase sigma factor	0	1	0	2
WRKY	14	0	16	0
Zinc finger	3	9	1	5

**Table 11:** Transcription factors that showed specific regulations in *A. scabra* or *A. stolonifera*

Name	Log2 FC	Species
High mobility group B protein 7	5.13	*A. scabra*
Dehydration-responsive element-binding protein 1A	3.43	*A. scabra*
Multiprotein-bridging factor 1c	2.69	*A. scabra*
Zinc finger CCCH domain-containing protein 47	2.28	*A. scabra*
Probable transcription factor GLK1	−1.84	*A. scabra*
GATA transcription factor 21	−1.97	*A. scabra*
Protein REVEILLE 1	−2.34	*A. scabra*
GATA transcription factor 26	−2.81	*A. scabra*
Trihelix transcription factor ASR3	−3.19	*A. scabra*
Transcription factor HY5	−4.16	*A. scabra*
Transcription factor DIVARICATA	5.44	*A. stolonifera*

### Validation of RNA-seq with qRT-PCR

**Fig. 10:**
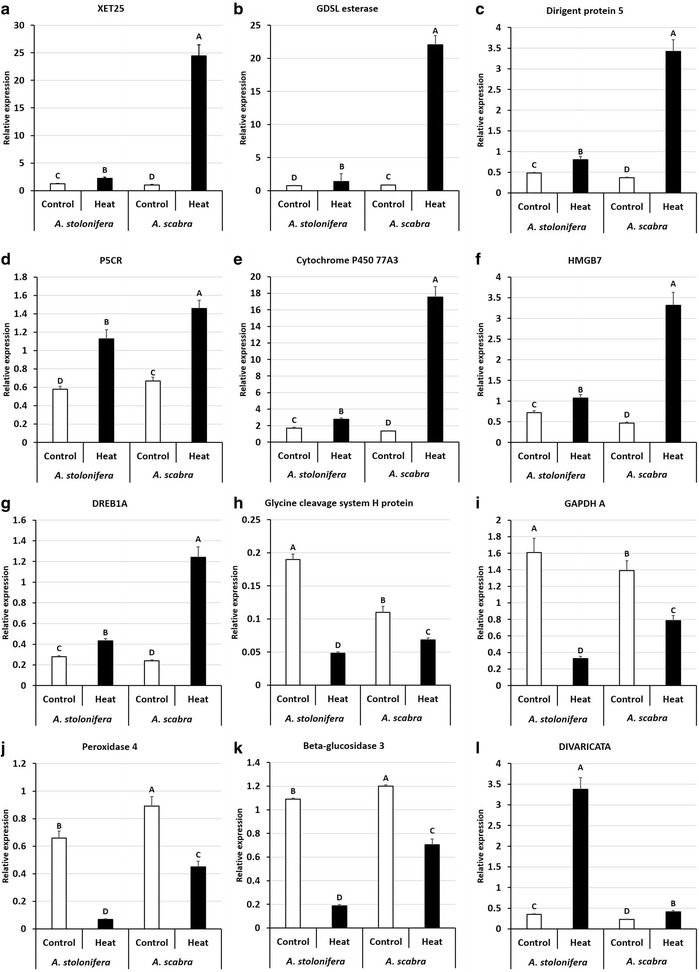
Relative gene expression levels of selected transcripts, including XET25 (**a**), GDSL esterase (**b**), Dirigent protein 5 (**c**), P5CR (**d**), Cytochrome P450 77A3 (**e**), HMGB7 (**f**), DREB1A (**g**), Glycine cleavage system H protein (**h**), GAPDH (**i**), Peroxidase 4 (**j**), Beta-glucosidase 3 (**k**), and DIVARICATA (**l**) in *A. stolonifera* and *A. scabra* under control and heat stress conditions by qRT-PCR. Data shown are the means of four biological replicates (*n* = 4). Bar represents standard error (SE) for each mean value. Different letters atop bars indicate that significant differences exist at P level < 0.05

The differential expressions of several DEGs in the RNA-seq data were validated using qRT-PCR. Heat stress significantly increased gene expression levels of xyloglucan endo-transferase 25 (XET25), GDSL esterase, Dirigent protein 5, pyrroline-5-carboxylate reductase (P5CR), Cytochrome P450 77A3, HMGB7, and DREB1A in both *A. stolonifera* and *A. scabra*. However, expression levels for these genes in *A. scabra* under heat stress were significantly higher than those in *A. stolonifera* (Fig. 10a-g). Heat stress significantly decreased gene expression levels of glycine cleavage system H protein, GAPDH A, peroxidase 4, and beta-glucosidase 3 in both *A. stolonifera* and *A. scabra*. However, the expression levels for these genes in *A. scabra* under heat stress were significantly higher than those in *A. stolonifera* (Fig. 10h-k). Heat stress also significantly increased expression level of transcription factor DIVARICATA in both *A. stolonifera* and *A. scabra*, but the expression level in *A. stolonifera* under heat stress was significantly higher than that in *A. scabra* (Fig. 10l). Furthermore, the fold changes of these genes obtained by qRT-PCR analysis were compared with RNA-seq data. The Person’s correlation coefficient between data from RNA-seq and qRT-PCR was 0.95 for *A. scabra*, and 0.93 for *A. stolonifera*, indicating that the transcriptional regulations under heat stress in these two species were valid regardless of detecting methods (Table 12).

**Table 12:** The qRT-PCR validation of selected genes in RNA-seq data

Gene	ID	Species	Log2FC in qPCR	Log2FC in RNA-seq
XET25	TRINITY_DN127707_c4_g25_i2	*A. scabra*	4.57	6.60
GDSL esterase	TRINITY_DN125263_c6_g4_i1	*A. scabra*	4.68	8.66
Dirigent protein 5	TRINITY_DN89062_c0_g1_i1	*A. scabra*	3.21	4.30
P5CR	TRINITY_DN120079_c1_g2_i1	*A. scabra*	1.12	1.82
Cytochrome P450 77A3	TRINITY_DN133782_c0_g2_i3	*A. scabra*	3.68	6.45
HMGB7	TRINITY_DN119330_c0_g1_i2	*A. scabra*	2.82	5.13
DREB1A	TRINITY_DN125656_c0_g3_i2	*A. scabra*	2.37	3.43
Pearson’s correlation (*A. scabra*)	0.95			
Glycine cleavage system H protein	TRINITY_DN88310_c1_g1_i3	*A. stolonifera*	−1.98	−2.49
GAPDH A	TRINITY_DN108728_c4_g45_i1	*A. stolonifera*	−2.31	−3.09
Peroxidase 4	TRINITY_DN101060_c1_g1_i1	*A. stolonifera*	−3.28	−9.13
Beta-glucosidase 3	TRINITY_DN113597_c1_g1_i1	*A. stolonifera*	−2.53	−8.22
DIVARICATA	TRINITY_DN89810_c0_g3_i1	*A. stolonifera*	3.27	5.44
Pearson’s correlation (*A. stolonifera*)	0.93			

## Discussion

The comparative analysis of transcriptome profiles between *A. stolonifera* and *A. scabra* exposed to heat stress found that metabolic processes involved in heat responses were similar in the two species, but some TFs and genes uniquely enriched in *A. scabra*, which could account for its superior heat tolerance. The following sections focus on the discussion of uniquely up-regulated TFs and genes in *A. scabra* and uniquely down-regulated TFs and genes in *A. stolonifera* regarding their functions and roles in heat tolerance.

Previous studies with *A. scabra* found that accumulation of carbohydrates and amino acids play major roles in heat tolerance for this heat-tolerant grass species [20, 22, 36, 37]. The GO term and KEGG analysis identified some specific pathways of genes involved in carbohydrate, amino acid, and energy metabolism, including unique up-regulation of glucosidases and monosaccharidases activity in *A. scabra* (Fig. 5, Table 6), and down-regulation of aminotransferase activity, glucose metabolic process, pentose phosphate shunt, transketolase, cis-trans isomerase, GAPDH, glycolate oxidase activity, cofactor binding (NAD, NADP, FMN), glucose-6-phosphate dehydrogenase and chloroplast organization in *A. stolonifera* (Fig. 8, Table 7). In addition, serine hydroxymethyltransferase 1 (SHMT1) was significantly down-regulated only in *A. stolonifera* (Table 7). SHMT catalyzes the interconversion between serine and glycine [38]. Previous study of root proteomic profiles between *A. stolonifera* and *A. scabra* under heat stress showed that one SHMT protein spot was decreased only in *A. stolonifera*, which agreed our transcriptional observation [19, 20]. In contrast to transcript responses of heat-sensitive *A. stolonifera*, lack of down-regulation of some genes mentioned above in *A. scabra* suggest that the maintenance of transcriptional levels of genes in carbohydrate, and amino acid, and energy metabolism may be associated with the corresponding metabolite accumulation under heat stress, contributing to the superior heat tolerance.

Under heat stress, *A. scabra* showed up-regulation of several functional categories that were related in antioxidative responses and antioxidant protection, while many of the functional categories related to oxidative protection, such as peroxidase activity, peroxisome, were down-regulated in *A. stolonifera* (Figs. 8 and 9). Most of the up-regulated antioxidant-related genes were Cytochrome P450s (Table 6). The cytochrome P450 is a superfamily catalyzing various oxidative reactions, including biosynthesis of lipophilic compounds (fatty acids, sterols, cutin, suberine and wax, phenylpropanoids, brassinolides and gibberellins [39]. The microarray analysis of cytochrome P450 family in Arabidopsis showed that they are highly responsive to both abiotic and biotic stresses [40]. Other up-regulated genes involved in antioxidant defense included oxidoreductase and monooxygenase activity found in *A. scabra* under heat stress, which were also mainly involved in plant antioxidative response. ROS content in *A. scabra* root tissue under heat stress was significantly lower than that in *A. stolonifera* [41], suggesting that the ROS scavenging capacity was better maintained in *A. scabra* under heat stress. It is also worthy to point out the 4-hydroxyphenylacetaldehyde oxime monooxygenase that were uniquely down-regulated in *A. stolonifera* was also a Cytochrom P450 gene (CYP71E1), which is involved in the oxime-metabolizing step in biosynthesis of dhurrin [42]. Little information was known about dhurrin and its relation to heat response in plants, which deserves further investigation.

Transcripts in proline biosynthesis, mainly pyrroline-5-carboxylate reductase (P5CR), were also up-regulated in *A. scabra* under heat stress (Fig. 4, Table 6). P5CR is the final step in proline biosynthesis pathway, which reduces proline-5-carboxylate to proline [43]. It is generally accepted that proline acts as a cellular osmolyte, and thus its accelerated biosynthesis indicates enhanced plant osmotic stress resistance [44]. In addition, proline is also involved in maintenance of redox balance and ROS scavenging [45–47]. Higher levels of proline were identified in heat-stressed cotton (*Gossypium hirsutum* L.) [48], and its positive role in heat tolerance was confirmed in various plant species [1, 49, 50]. Our previous study found that proline content was significantly higher in *A. scabra* root tissues under heat stress than that in *A. stolonifera* [22]. However, there is little information regarding to P5CR expression regulation under heat stress. This study found the up-regulation of pyrroline-5-carboxylate reductase in *A. scabra,* which could play positive roles in the maintenance of proline synthesis in the heat-tolerant species under heat stress.

Most of the transcripts up-regulated in lipid catabolic process were GDSL esterases and Phospholipase A1 (Table 6). The GDSL-motif enzyme is a newly discovered lipase family that shares the highly conserved motif Gly-X-Ser-X-Gly (X means any amino acid) in the sequence [51, 52]. The number of GDSL esterase/lipase family members ranged from 57 to 130 in several plant organisms [53, 54]. The GDSL esterases/lipases might play an important role in plant development and morphogenesis [52]. Some of the GDSL esterases were reported to confer plant abiotic stress tolerance, such as drought and salt stress [55, 56]. Phospholipase A1 is one of the multigene family of phospholipases, hydrolyzing the sn-1 acylester bond of phospholipids to free fatty acids and 2-acyl-1-lysophospholipids [57]. Compared to mammalian phospholipase A1s, only a few genes were discovered in plants. A phospholipase A1 homolog in Arabidopsis, AtDAD1, was placed in the initial step of jasmonic acid biosynthesis, making it important for plant responses to abiotic stress, tendril coiling, fruit ripening and developmental maturation of stamens and pollens [58]. Another phospholipase A1 in hot pepper (*Capsicum annuum*) showed high sequence similarity to Arabidopsis [59]. Another phospholipase A1 homolog in Arabidopsis, AtLCAT3, was determined to have in vitro enzymatic activity, although its molecular function has yet to be assigned [60]. Therefore, the GDSL esterases and phospholipase A1s found in the up-regulated transcripts in *A. scabra* were also considered to be involved in lipid catalysis, possibly through jasmonic acid signal transduction pathway. This is first report of GDSL esterases and phospholipase A1s related to heat tolerance. Further studies regarding to their functions and regulation of heat tolerance in plants are needed.

The transcripts involved in cell wall structure and properties were up-regulated in *A. scabra* under heat stress, including xyloglucan endo-transglycosylases (XETs), and cellulose synthase (Table 6). XETs make nonhydrolytic cleavage and ligation of xyloglucan chains, which is involved in cell wall loosening [61]. Cellulose synthase family is also well-defined, and involved in the formation of plant primary and secondary cell wall [62] Plant cell wall structure undergoes reassembly that involves biosynthesis of major cell wall components during plant responses to abiotic stress [63–65]. Xu et al. [66] reported that transcript levels of XETs in tall fescue root tissues were decreased under water stress, and exogenous application of ascorbic acid could mitigate the reduction. Little information was known regarding to genes for cell wall biosynthesis and properties related to heat tolerance.

Several transcripts involved in lignan biosynthetic process were also up-regulated in *A. scabra*, including dirigent protein 5, aureusidin synthase 1, and (+)-larreatricin hydroxylase 1 (Table 6). Dirigent proteins play an important role in monolignol coupling to both lignin and lignan formations [67]. Dirigent protein family was reported to participate in defense responses, secondary metabolism, temperature, and salinity stress [68–70]. Aureusidin synthase is a binuclear copper enzyme, and a homolog of plant phenol oxidase [71]. It is proposed to be a chalcone-specific plant phenol oxidase for aurone biosynthesis [72]. Larreatricin hydroxylase is an enantio-specific polyphenol oxidase [73], but their physiological roles in plant adaptation to abiotic stress are unknown. Results in our study indicated that the up-regulation of genes involved in secondary cell-wall materials could contribute to the maintenance of cell wall structure and functional properties for *A. scabra* to maintain growth under heat stress.

Several transcription factors were uniquely up-regulated under heat stress, including high mobility group B protein (HMGB) 7, dehydration-responsive element-binding factor (DREB) 1a, Multiprotein-bridging factor (MBF) 1c, and CCCH-type zinc finger protein 47 (Table 11). The high mobility group B protein (HMGB) belongs to chromatin-associated proteins, and acts primarily as architectural facilitator in nucleoprotein complex assembly and transcriptional regulation and recombination [74, 75]. Little is known about its function in plant stress responses, except that Arabidopsis HMGBs showed induced expression levels under cold stress [76]. Dehydration-responsive element-binding factor (DREB) is one of the sub-groups in AP2/EREBP family, and activates target genes that have dehydration-responsive elements (DREs) [77, 78]. DREB1 and DREB2 were reported to confer plant drought, salinity and low-temperature tolerance [79–81], while only Arabidopsis DREB2A has dual functions in water-stress and heat-stress response [82]. Multiprotein-bridging factor 1 (MBF1) is a conserved transcriptional coactivator that bridges a basic region/leucine zipper (bZIP) type coactivator and a TATA-box binding protein [83, 84]. The Arabidopsis MBF1c was induced in response to heat stress [84, 85]. The transgenic Arabidopsis constitutively expressing MBF1c enhanced plant heat tolerance by perturbing ethylene response signal transduction pathway [86]. Plant CCCH-type zinc finger proteins were shown to be involved in embryo formation, floral reproductive organ formation, delay of leaf senescence, and calmodulin-mediated RNA processing [87–90]. Two CCCH-type zinc finger proteins, AtSZF1 and AtSZF2, were induced upon salt stress, and negatively regulate salt-responsive genes in Arabidopsis [91]. Our results suggest that the up-regulation of HMGB7, DREB1a, MBF1c, and CCCH-type zinc finger protein 47 could active the related down-stream genes regulating heat tolerance. Further research is needed to identify the down-stream genes in order to unravel the molecular roles of those transcriptional factors in heat tolerance.

## Conclusions

**Fig. 11:**
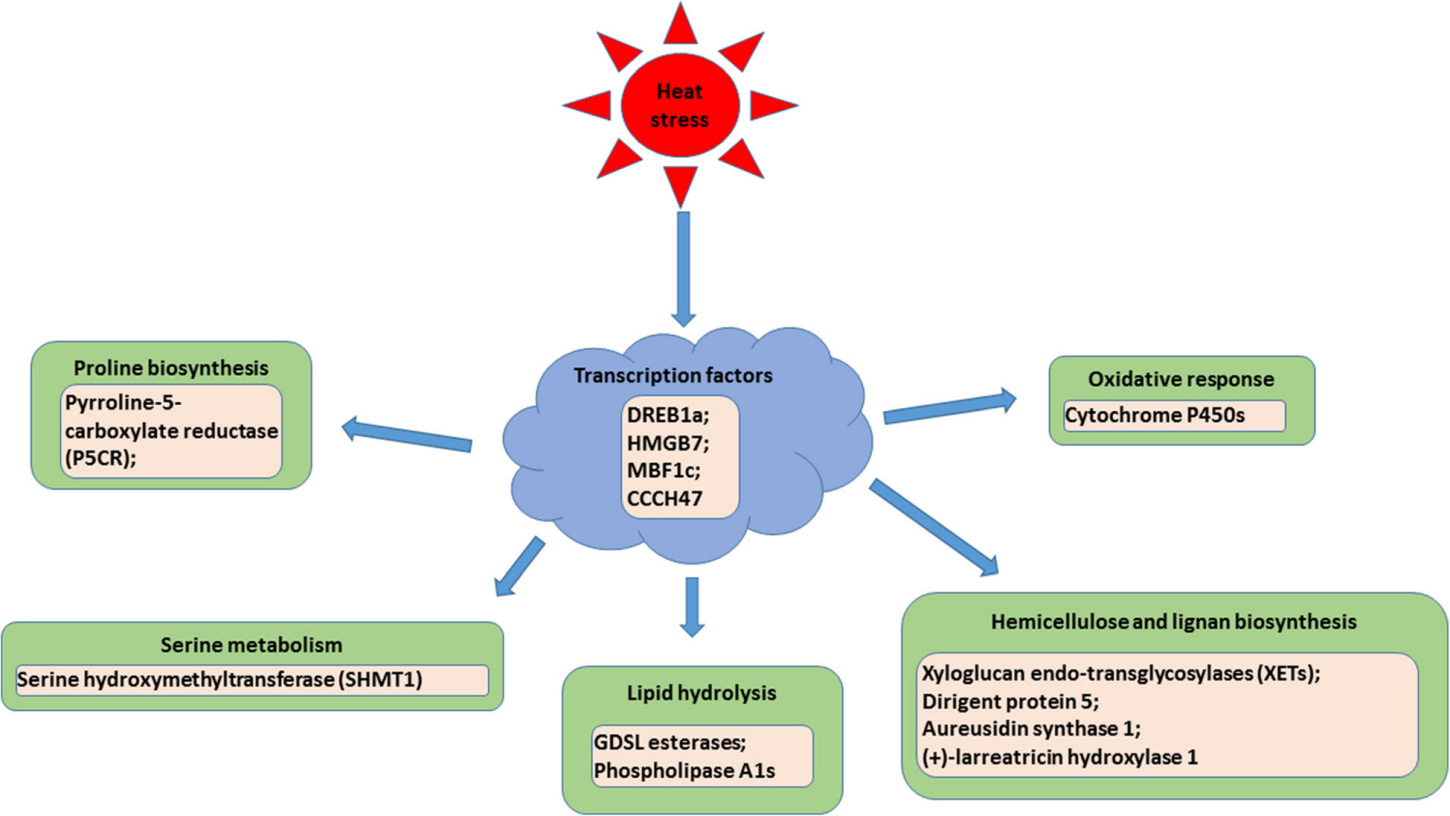
Summary and proposed pathways for transcriptional regulation of heat tolerance in *Agrostis* grass species

In summary, our comparative analysis of transcriptomic changes in response to heat stress for heat-tolerant thermal *A. scabra* and heat-sensitive *A. stolonifera* showed divergent transcriptional regulations of heat tolerance in perennial grass species, which complemented to previous findings of physiological traits and proteins conferring the superior heat tolerance of the thermal species adapted to extremely high soil temperature. The potential novel transcriptional regulatory mechanisms for the superior heat tolerance in thermal *A. scabra* plants are proposed based on the results described above (Fig. 11). Heat stress could trigger molecular responses in *A. scabra* by up-regulating TFs, such as high mobility group B protein 7 (HMGB7), dehydration-responsive element-binding factor 1a (DREB1a), multiprotein-bridging factor 1c (MBF1c), CCCH-domain containing protein 47 (CCCH47), and downstream genes involved in serine metabolism (serine hydroxymethyltransferase, SHMT1), oxidative protection (cytochrome P450s), proline biosynthesis (pyrroline-5-carboxylate reductase, P5CR), lipid hydrolysis (GDSL estarases, phospholipase A1), hemicellulose and lignan biosynthesis (xyloglucan endo-transglycosylases, XETs and dirigent protein 5, aureusidin synthase 1, and (+)-larreatricin hydroxylase 1). The direct relationship and roles of those uniquely-expressed TFs and genes in heat-tolerant *A. scabra* than those in heat-sensitive *A. stolonifera* requires further confirmation.

## Additional files

Additional file 1: Heat map of GO term enrichment analysis for up-regulated DEGs in *A. stolonifera* (P) and *A. scabra* (N). Scale represents log10 of *P*-value in the enrichment analysis. (JPEG 575 kb)

Additional file 2: Heat map of GO term enrichment analysis for down-regulated DEGs in *A. stolonifera* (P) and *A. scabra* (N). Scale represents log10 of *P*-value in the enrichment analysis. (JPEG 3438 kb)

## Abbreviations

AP2/ERF: APETALA2/ethylene-responsive element binding factor
CCCH: CCCH-domain containing protein
DEG: Differentially expressed gene
DREB: Dehydration-responsive element binding factor
DW: Dry weight
EL: Electrolyte leakage
FDR: False discovery rate
FW: Fresh weight
GO: Gene ontology
HMGB: High mobility group B protein
HSF: Heat shock factor
MBF: Miltiprotein-bridging factor
MYB: Myeloblastosis factor
P5CR: Pyrroline-5-carboxylate reductase
PAR: Photosynthetically active radiation
RH: Relative humidity
RWC: Relative water content
SHMT: Serine hydroxymethyltransferase
TF: Transcription factor
TW: Turgid weight
WRKY: WRKY-domain factor
XET: Xyloglucan endo-transferase
