# Supplementary figures and images for: Transcriptomic analysis reveals unique molecular factors for lipid hydrolysis, secondary cell-walls and oxidative protection associated with thermotolerance in perennial grass
Source: BMC Genomics. 2018 Jan 22;19:70. doi: 10.1186/s12864-018-4437-z (PMC5778672; doi:10.1186/s12864-018-4437-z)

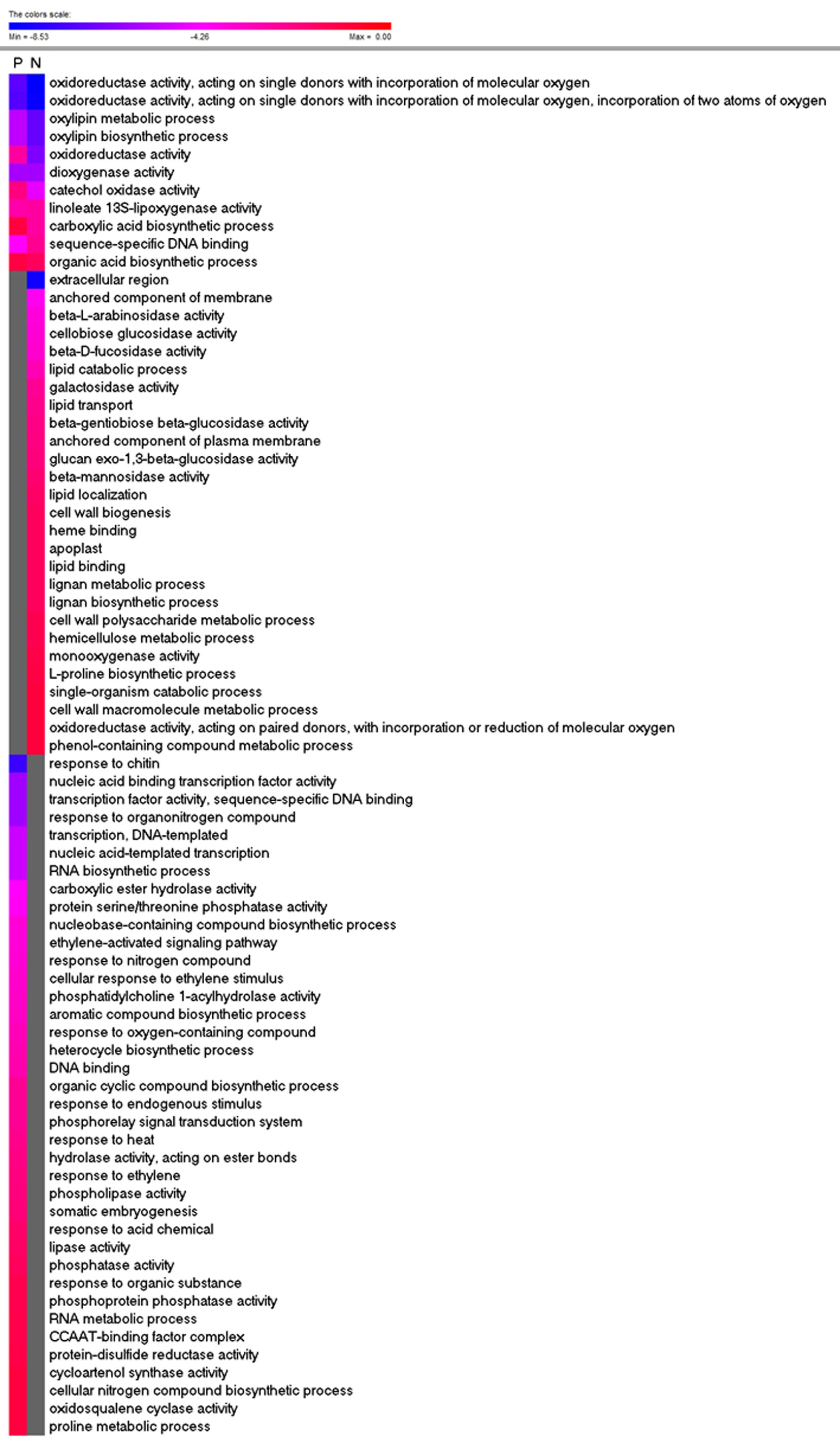

Supplement: Supplementary file 1 — Heat map of GO term enrichment analysis for up-regulated DEGs in A. stolonifera (P) and A. scabra (N). Scale represents log10 of P-value in the enrichment analysis. (JPEG 575 kb) [file 12864_2018_4437_MOESM1_ESM.jpg]

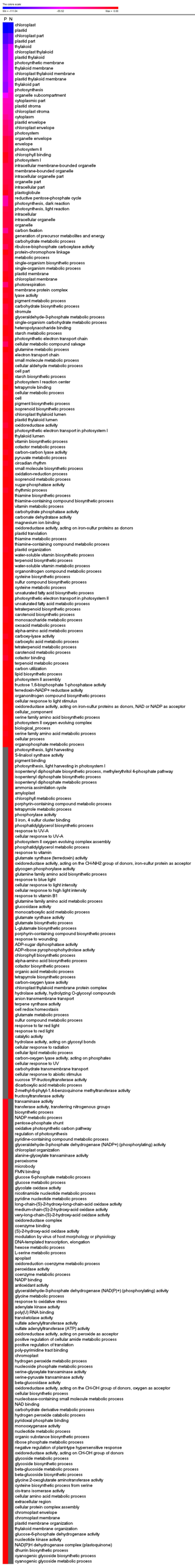

Supplement: Supplementary file 2 — Heat map of GO term enrichment analysis for down-regulated DEGs in A. stolonifera (P) and A. scabra (N). Scale represents log10 of P-value in the enrichment analysis. (JPEG 3438 kb) [file 12864_2018_4437_MOESM2_ESM.jpg]
